# Rapid Evaluation in Whole Blood Culture of Regimens for XDR-TB Containing PNU-100480 (Sutezolid), TMC207, PA-824, SQ109, and Pyrazinamide

**DOI:** 10.1371/journal.pone.0030479

**Published:** 2012-01-18

**Authors:** Robert S. Wallis, Wesley Jakubiec, Mark Mitton-Fry, Lynn Ladutko, Sheldon Campbell, Darcy Paige, Annette Silvia, Paul F. Miller

**Affiliations:** 1 Pfizer, Groton, Connecticut, United States of America; 2 VA Connecticut Healthcare, West Haven, Connecticut, United States of America; 3 Yale University, New Haven, Connecticut, United States of America; National Institute of Immunology, India

## Abstract

There presently is no rapid method to assess the bactericidal activity of new regimens for tuberculosis. This study examined PNU-100480, TMC207, PA-824, SQ109, and pyrazinamide, singly and in various combinations, against intracellular *M. tuberculosis*, using whole blood culture (WBA). The addition of 1,25-dihydroxy vitamin D facilitated detection of the activity of TMC207 in the 3-day cultures. Pyrazinamide failed to show significant activity against a PZA-resistant strain (*M. bovis* BCG), and was not further considered. Low, mid, and high therapeutic concentrations of each remaining drug were tested individually and in a paired checkerboard fashion. Observed bactericidal activity was compared to that predicted by the sum of the effects of individual drugs. Combinations of PNU-100480, TMC207, and SQ109 were fully additive, whereas those including PA-824 were less than additive or antagonistic. The cumulative activities of 2, 3, and 4 drug combinations were predicted based on the observed concentration-activity relationship, published pharmacokinetic data, and, for PNU-100480, published WBA data after oral dosing. The most active regimens, including PNU-100480, TMC207, and SQ109, were predicted to have cumulative activity comparable to standard TB therapy. Further testing of regimens including these compounds is warranted. Measurement of whole blood bactericidal activity can accelerate the development of novel TB regimens.

## Introduction

New drugs and regimens are urgently needed that can shorten the required duration of tuberculosis treatment. This need is greatest for extensively-drug resistant disease (XDR-TB), which presently requires at least 24 months of treatment for cure [1]. The requirement for prolonged therapy arises due to the persistence of dormant or semi-dormant mycobacteria that exhibit phenotypic drug tolerance [2]. Such subpopulations can arise solely by chance; however, their numbers are greatly amplified in response to oxygen or nutrient starvation *in vitro*, or to pressure from host immune mechanisms *in vivo*.

The problem of microbial persistence due to phenotypic tolerance is not unique to mycobacteria [3]. In streptococcal and enterococcal endocarditis, small numbers of bacteria can similarly persist and cause relapse unrelated to the acquisition of new drug resistance. Specific drug combinations – β-lactams plus aminoglycosides – increase the likelihood of endocarditis cure and shorten the required duration of treatment [4]. The goal of the present study was to identify similarly favorable new drug combinations for tuberculosis.

Current strategies to test new TB drug combinations have important shortcomings. MIC testing, a common first step, does not adequately predict bactericidal activity for TB drugs, even when comparisons are limited to drugs within a single class [5]. Short term studies in the mouse model, a common next step of testing, do not adequately predict sterilizing capacity [6]. This project instead used whole blood culture to rapidly examine the potential activity of new regimens. Mycobacteria added to heparinized blood are quickly ingested by phagocytic cells, first by neutrophils and subsequently by monocytes [7], [8]. Expression of host effector mechanisms results in inhibition of mycobacterial growth. The extent of inhibition depends on strain virulence and donor immune status [7], [9], being greater in tuberculin skin test positive donors or following vaccination with *M. bovis* Bacillus Calmette-Guérin (BCG), and reaching bacteriostasis in TB patients tested with their own isolates [10].

Several studies have examined the whole blood bactericidal activity (WBA) of TB drugs at multiple time points after oral drug administration. First line drugs show a rank order of cumulative WBA (its area under the curve [AUC] over 24 hrs) of rifampin>isoniazid>pyrazinamide>ethambutol when tested in healthy volunteers [8]. Current regimens for multi-drug resistant (MDR) disease are less active than standard therapy [11]. In one study of patients with drug-sensitive TB, cumulative WBA was greater during the intensive phase of standard therapy, correlated with the rate of decline of sputum log colony counts, and was superior in patients whose sputum cultures converted to negative after 8 weeks of treatment [10]. WBA may therefore be described as an emerging biomarker for TB treatment. Its predictive value may in part arise due to its ability to model drug effects against semi-dormant mycobacteria in the context of a bacteriostatic host immune response.

## Methods

**Figure 1:**
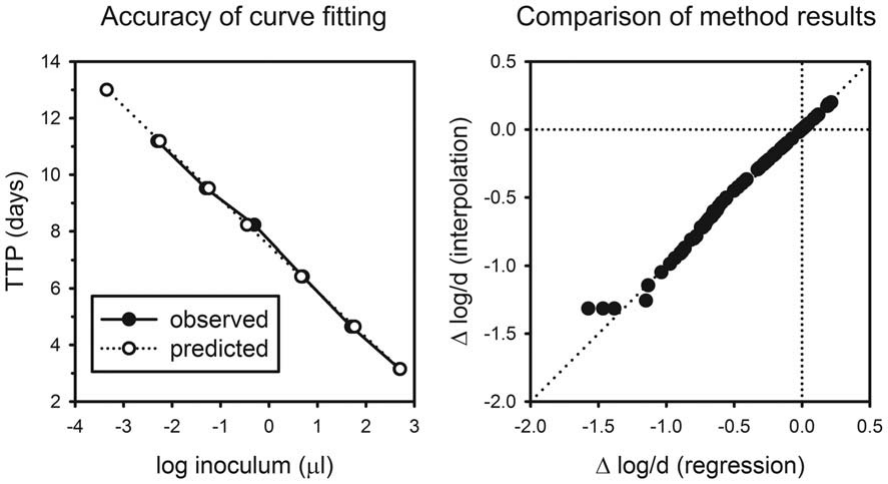
Effects of substituting regression analysis for point-to-point interpolation in calculating Δ log CFU from time to positivity (TTP) in mycobacterial growth indicator tubes (MGIT). The left panel indicates the accuracy of curve fitting (the relationship between observed and predicted values). The right panel compares the results of the two methods (regression vs. interpolation).

A single healthy volunteer served as a blood donor for this study, after providing written informed consent. The study protocol was approved by the Institutional Review Board of the Veterans Administration Connecticut Healthcare System. Drug powders were either dissolved first in dimethylsulfoxide (PNU-100480 [sutezolid], PA-824, TMC207 [bedaquiline], and SQ109), or RPMI-1640 tissue culture medium (isoniazid, rifampin, and pyrazinamide). Stock solutions were prepared at concentrations ranging from 10 mg/L (isoniazid) to 200 mg/L (pyrazinamide) and kept at −30°C until needed. WBA was measured as previously described [12] with exceptions as noted below. Briefly, *M. tuberculosis* H37Rv was grown in mycobacterial growth indicator tubes (MGIT, Becton-Dickinson) until 1–2 days after growth was detected, and frozen in aliquots. A titration experiment determined the relationship between inoculum size and time-to-positivity (TTP) over a 6-log range (500 µl to 0.005 µl, figure 1) using a MGIT 960 detection system, and identified the volume positive in 5.5 days. Whole blood cultures were conducted in sealed screw-top tubes with slow constant rotation. The cultures consisted of 300 µl heparinized blood, an equal volume of RPMI 1640 tissue culture medium, and the specified volume of mycobacterial stock. After 72 hrs, cells were sedimented, the liquid phase removed, and blood cells disrupted by hypotonic lysis. Bacilli were recovered, resuspended in 7H9 broth, and inoculated into MGIT. Days and hours until cultures were detected as positive were recorded. All cultures were performed in duplicate; TTP values were represented as the mean of duplicate cultures. Log change in viability was calculated as log(*final*) – log(*initial*), where *final* and *initial* are the volumes corresponding to TTP of the completed cultures and its inoculum, respectively, based on the titration curve. The laboratory protocol and software (version 12) developed by the author (RSW) are available on request. WBA was reported as log change/day.

**Figure 2:**
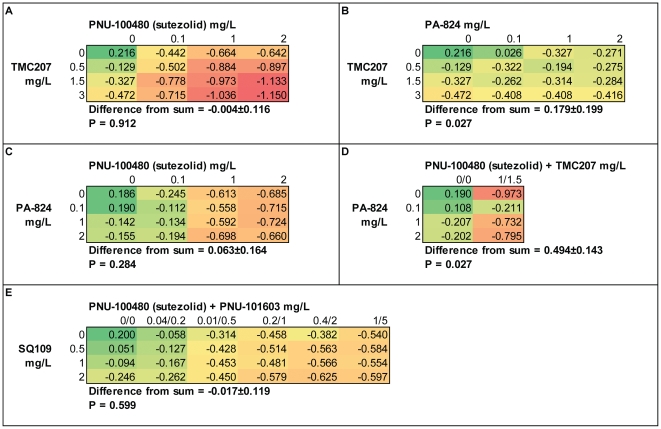
Bactericidal activities of 2 and 3-drug combinations against intracellular *M. tuberculosis* in whole blood culture. Negative values in these blocks indicate killing. A color gradient has been applied to indicate green as bacterial growth, and red as death. The difference from sum was determined by subtracting the activity predicted as the sum of both compounds tested individually from that observed when tested in combination. Positive values here indicate combinations less active than predicted. TMC207 and SQ109 showed additive activity when combined with PNU-100480. Combinations of PA-824 with either PNU-100480 or TMC207, or as a 3-drug combination, were less than additive.

Each drug was tested individually to establish its concentration – activity relationship. Drug pairs were then tested in a checkerboard fashion (figure 2). The observed activity of each drug pair was compared to that predicted by their sum when tested separately. The mean difference between observed and expected values for the full range of expected therapeutic concentrations was then calculated for each drug pair.

**Figure 3:**
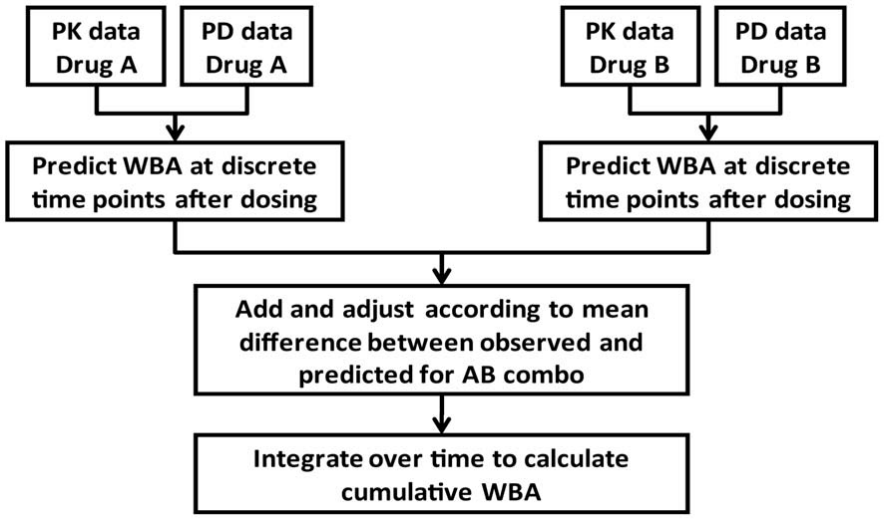
Process for calculating cumulative whole blood bactericidal activity (WBA) of drug combinations.

Drug activity after oral administration was predicted based on published PK data and the observed concentration-activity relationship (figure 3). For PA-824, mean values of 3 experiments were used. Drug concentrations *in vivo* are twice those in *ex vivo* whole blood culture, due to dilution with tissue culture medium. Expected concentrations in plasma of the novel compounds were therefore reduced by half so that results could be compared to orally-administered therapies. Values for hourly time points were estimated by interpolation. The combined activities of specific drug combinations were calculated for each hourly time point by adding the activities of the individual drugs and adjusting the result according to the mean difference between observed and predicted values. Cumulative WBA was then calculated as the AUC_0–24_ by the trapezoidal method, and reported as log change.

Paired values were compared using the paired t test. Comparisons among multiple values were performed by the repeated measures one-way analysis of variance (ANOVA). Post-hoc testing to examine differences between specific pairs was then performed by the Holm-Sidak method. All statistical tests were performed using SigmaPlot version 11.

## Results

Preliminary experiments addressed 3 concerns of this project: the method of determining the titration curve, the suitability of whole blood culture conditions to detect effects of TMC207 (bedaquiline), and the activity of pyrazinamide against pyrazinamide-resistant mycobacteria. Initial experiments had indicated that the time-to-positivity of MGIT cultures from highly bactericidal drug combinations could exceed those tested in the titration experiment. Such results cannot be analyzed by point-to-point interpolation, the method used in previous studies of this method. The WBA spreadsheet was therefore modified to perform exponential regression analysis rather than interpolation, using the equation y = y0+ae^−bx^ for curve fitting, where y = time-to-positivity in days, x = log inoculum volume in µl, and e = Euler's number. Values for y0, a, and b that most closely fit experimental results were identified using the iterative non-linear regression module (Solver) provided with Microsoft Excel, using its default parameters. This exponential decay equation was selected because it provided the best fit for all of 5 different batches of *M. tuberculosis* stock (not shown). The adequacy of curve fitting for the batch used in this study is illustrated by the superimposition of curves for predicted and observed data in the left panel of figure 1. The right panel of figure 1 compares assay results obtained using the two methods (regression *vs*. interpolation). The two sets of results are equal except for those samples showing maximal bactericidal effect, the activities of which were underestimated by the interpolation method. The exponential regression method was therefore selected for use in this study.

A second preliminary experiment sought to identify whole blood culture conditions that adequately detected the effect of TMC207 (bedaquiline). This compound, which acts by selective inhibition of mycobacterial ATP synthetase [13], has a delayed time to onset *in vivo* and *in vitro* that presumably reflects the size of the ATP pool at the start of drug exposure and the net balance between ATP consumption and production. Studies in patients treated with TMC207 indicate a 4-day delay before an effect on mycobacterial viability in sputum becomes evident [14]. Shorter times to onset of action of TMC207 have been reported under hypoxic conditions [15] and in intracellular infection [16] as compared to aerobic broth culture. However, no previous studies have examined the effects of TMC207 in cell culture infection models of 3-day duration. Mammalian cell viability in the sealed whole blood cultures is limited by progressive depletion of oxygen and nutrients. We therefore tested conditions with different head space volumes and culture durations to determine effects on drug activity (table 1). We included cultures in which 15 nM 1,25-dihydroxy vitamin D was added, based on the hypothesis that stresses due to activation of cellular anti-mycobacterial mechanisms by vitamin D [17] might in turn accelerate mycobacterial ATP consumption and thereby hasten onset of drug action. Negative values in table 1 indicate bactericidal activity. The addition of vitamin D indeed enhanced our ability to detect the effect of TMC207 without requiring changes in whole blood culture tube geometry or duration. The vitamin D concentration was insufficient to have a direct effect on *M. tuberculosis* viability. All subsequent experiments were therefore conducted in the presence of added vitamin D.

**Table 1:** Bactericidal activities of TMC207, PNU-1004800, rifampin (RIF), isoniazid (INH), and pyrazinamide (PZA), against intracellular *M. tuberculosis* in sealed whole blood cultures differing according to duration, air space, and addition of dihydroxy-vitamin D.

Vitamin D	Culture duration	Culture volume air/liquid	nil	TMC207 1.5 mg/L	PNU-100480 1 mg/L	RIF 5 mg/L	INH 1 mg/L	PZA 20 mg/L
*(nM)*	*(days)*	*(ml)*	*Δ log CFU/d*					
0	3	0.1/0.4	0.086	0.117	−0.745	−1.382	−0.936	0.048
0	3	1.4/0.6	−0.004	0.022	−0.652	−1.575	−0.817	0.195
0	7	1.4/0.6	0.182	0.180	−0.299	−1.034	−0.562	0.143
15	3	0.1/0.4	−0.022	−0.073	−0.864	−1.466	−0.936	0.009
15	3	1.4/0.6	0.216*	−0.327*				

Negative values indicate killing.

*Results are carried over from figure 2.

A final preliminary experiment examined the activity of pyrazinamide-containing regimens against PZA-resistant *M. bovis* BCG (ATTC TMC 1101). Although pyrazinamide has historically been a key sterilizing TB drug, recent surveys indicate resistance rates as high as 85% in MDR-TB strains [18]. PZA is often empirically included in regimens for M/XDR-TB, due to uncertainties as to optimal methods for resistance testing. One study has suggested that pyrazinamide may act at least in part via a host cellular target [19], thus casting doubt on the significance of microbial pyrazinamide resistance. *M. bovis* strains (including BCG) are characteristically resistant to pyrazinamide [20]. Unlike *M. tuberculosis*, which grew in the absence of added drug, viability of the attenuated BCG strain declined by log −0.135. Progressive addition of vitamin D, PNU-100480, and PZA (20 mg/L) resulted in progressive loss of viability, to log −0.151, −0.372, and −0.410, indicating a net incremental effect of pyrazinamide of −0.038. The effect of pyrazinamide appeared substantially reduced compared that when tested against *M. tuberculosis* following oral administration (mean, −0.100±.065) [12]. Given the widespread reports of PZA-resistance in M/XDR-TB, and our inability to show strong activity against PZA-resistant strains, pyrazinamide was not considered further for inclusion in candidate regimens against XDR-TB.

The bactericidal activities of drugs tested individually and in 2 and 3-way combinations are shown in figure 2. Drug concentrations were selected to approximately reflect low, mid, and high therapeutic plasma concentrations following oral dosing [12], [14], [21]. A color gradient has been applied across all cells to indicate green as bacterial growth, and red as death. The activities of individual compounds appear in the first column and row of each checkerboard. When tested individually, the activities of TMC207 and SQ109 increased progressively with increasing drug concentration. This was not the case for either PNU-100480 (sutezolid) or PA-824, which showed no further increase in activity as concentrations increased from mid to high therapeutic levels. Within the range of tested concentrations, PNU-100480 was the most active single drug, differing from the others by one way repeated measures ANOVA (P = .004) and post-hoc analysis (Holm-Sidak method).

Cells other than those in the first column and row in figure 2 indicate the combined effects of multi-drug combinations. The combinations differed significantly by one way repeated measures ANOVA (P<.001). PNU-100480 plus TMC207 was the most active combination, and PA-824 plus TMC207, the least.

For each combination, the observed activity was compared to that expected as the sum of each drug tested individually. The mean and standard deviation of the difference between observed and expected values appears in each panel. Positive values indicate drug combinations that overall were less than additive, and negative values, more than additive. No combination was significantly more than additive. The combinations of PNU-100480 plus TMC207 (panel A) and PNU-100480 plus SQ109 (panel E) overall were additive, each having observed and expected values that did not differ significantly. In contrast, the combination of PA-824 plus TMC207 (panel B) was significantly less active than expected (mean difference = +.179, P = .027 by paired t test). For PA-824 plus PNU-100480 (panel C), a trend toward reduced activity overall was present (mean difference = +.063, P = .284). Panel D shows the predicted and actual activity of the PNU-TMC-Pa combination. In this case, limited testing was performed, using only mid concentrations of PNU-100480 and TMC207. Despite the reduced number of test conditions, the combination was significantly less active than predicted (difference = +.494, P = .027). Inspection of individual cells revealed antagonism when low concentrations of PA-824 were combined with low or mid concentrations of PNU-100480. Under these conditions, the activity of the two drugs combined appeared less than that of PNU-100480 alone. Antagonism was also apparent when low concentrations of PA-824 were combined with mid concentrations of TMC207 plus PNU-100480.

For 3 of the compounds tested (PNU-100480, TMC207, and PA-824), knowledge of a compound's concentration-activity relationship and pharmacokinetics in plasma should enable the prediction of its cumulative WBA after oral dosing according to the method outlined in figure 3. This hypothesis was tested for PNU-100480 at a dose of 600 mg orally BID, for which both PK and WBA were previously measured in 7 healthy volunteers [12]. To calculate predicted WBA from *in vitro* data, drug concentrations in whole blood culture was assumed to be half those in blood, due to dilution with tissue culture medium. Predicted cumulative WBA in these subjects (−0.367±0.065, mean SD) did not differ significantly from that measured directly (−0.316±0.044) according to paired t test (P = .06). However, the trend toward statistical significance indicated that cumulative WBA *ex vivo* might be lower than that predicted *in vitro*. We therefore adopted a conservative approach and used the *ex vivo* values for PNU-100480 for all subsequent comparisons. In each case, the activity of a regimen was predicted as the sum of the individual drugs adjusted by the mean difference between observed and predicted values for that combination.

**Figure 4:**
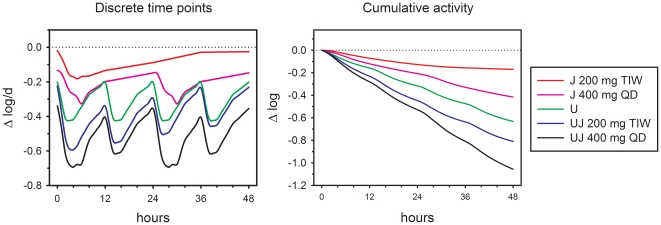
Predicted whole blood bactericidal activity of TMC207 (J, bedaquiline) 400 mg QD and 200 mg TIW, and PNU-100480 (U, sutezolid) 600 mg BID, singly and together.

Results for combinations of PNU-100480 and TMC207 are illustrated in figure 4. Current recommended dosing for TMC207 is 400 mg daily for the first 2 weeks of treatment, followed by 200 mg thrice weekly (TIW) thereafter [14]. Plasma levels of TMC207 are lower during intermittent treatment. This is reflected in the reduced cumulative WBA during intermittent *vs*. daily dosing, whether given individually, or in combination with PNU-100480.

Results for other 2, 3, and 4-drug combinations are summarized in table 2. In the case of SQ109, drug activity in the lung cannot be directly estimated based on levels in plasma. In the mouse, SQ109 concentrations in lung are more than 100-fold greater than those in plasma, reaching concentrations >10-fold above MIC [22]. The data for SQ109 in table 2 assume a mean effective concentration of 2 mg/L, 4-fold above MIC. At this concentration, SQ109 adds cumulative bactericidal activity of log−0.14 to that of PNU-100480. This is consistent with other reports of favorable interactions of SQ109 with both TMC207 and PNU-100480 in various *in vitro* models [23], [24]. The combination of PNU-100480, TMC207, and SQ109 was the most active of those evaluated. It compared favorably to cumulative WBA reported in TB patients receiving standard daily therapy, with a value intermediate to those during the intensive and continuation phases of treatment [10]. Readers will note that in the 2001 and 2003 publications, WBA was reported as log change over the entire 3 day period of whole blood culture rather than as log change per day. Those values have here been reduced by a factor of 3 for consistency with current methods.

**Table 2:** Predicted cumulative whole blood bactericidal activity of single drugs and multi-drug combinations against *M. tuberculosis*, based on published pharmacokinetic data, and the concentration-activity relationships and drug-drug pharmacodynamic interactions in figure 2.

	Cumulative WBA
**Single drugs**	***Δ log***
Pa 200 mg QD	−0.09
J 200 mg TIW	−0.09
Pa 600 mg QD	−0.22
J 400 mg QD	−0.21
U 600 mg BID*	−0.32
**2-drug combinations**	
Pa 200 mg QD+J 200 mg TIW	+0.01
Pa 600 mg QD+J 400 mg QD	−0.25
U*+Pa 200 mg QD	−0.34
U*+Pa 600 mg QD	−0.47
U*+J 200 mg TIW	−0.41
U*+J 400 mg QD	−0.53
U*+Sq	−0.46
**3-drug combinations**	
U*+J 200 mg TIW+Pa 200 mg QD	−0.00
U*+J 400 mg QD+Pa 600 mg QD	−0.25
U*+Sq+J 200 mg TIW	−0.55
U*+Sq+J 400 mg QD	−0.67
**4-drug combinations**	
U*+Sq+J 200 mg TIW+Pa 200 mg QD	−0.24
U*+Sq+J 400 mg QD+Pa 600 mg QD	−0.50
**Comparators from published literature**	
H*+R*+Z* (tested against MDR isolates) [8]	−0.02
L*+E*+Z* (tested against MDR isolates) [8]	−0.23
H*^+^+R*^+^ [10]	−0.59
H*^+^+R*^+^+E*^+^+Z*^+^ [10]	−0.77

Drugs were tested by direct adding drugs directly to whole blood cultures of healthy volunteers except for: * drugs were administered orally; ^+^ subjects were TB patients. U = PNU-100480 600 mg BID; J = TMC207; Pa = PA-824; H = isoniazid; R = rifampin; E = ethambutol; Z = pyrazinamide; L = levofloxacin. The combination of PNU-100480, SQ109, and TMC207 400 mg QD was the most active of those tested.

Antagonism was apparent when 200 mg PA-824 was added to some PNU or TMC-containing regimens. Of the five instances in table 2 in which 200 mg PA-824 was added to a drug or drug combination, four were predicted to show antagonism (*i.e.*, reduced activity with PA-824 added). At the 600 mg dose, two of four showed antagonism. The remainder all showed less than additive effects.

## Discussion

This study examined the bactericidal activities of PNU-100480, TMC207, PA-824, and SQ109, singly and in various combinations, using whole blood culture. These compounds are each members of new classes of antimycobacterial drugs: oxazolidinone, diarylquinoline, nitroimidazole, and ethylenediamine, respectively. None exhibits cross-resistance with existing TB drugs. For each, MICs against M/XDR-TB strains equal those for fully drug-sensitive strains. As a result, new regimens comprised of these drugs will be as active against highly resistant mycobacterial strains as for drug sensitive strains. This permits experimental studies with a fully sensitive *M. tuberculosis* strain such as H37Rv to inform activity against highly resistant strains [25].

The main findings of the present study were that combinations of PNU-100480 and TMC207 were highly active, approaching the combined activity of isoniazid plus rifampin, whereas 2 and 3-drug combinations including PA-824 showed less than fully additive activity. These observations can provide important guidance in the selection for further testing of novel regimens against tuberculosis in animal models and in patients. At the same time, the findings raise questions as to the mechanisms underlying these observations, and the extent to which *in vitro* models can replicate the complexity and variability of human disease.

The range of drug concentrations tested in this study reflected those in plasma in phase 1 and 2 clinical trials, recognizing that our understanding of optimal dosing of these drugs may yet be incomplete. PNU-100480 and PA-824 both have been reported to show time-dependent killing [12], [26]. The finding in the present study that neither drug showed increased activity as drug concentrations increased from 1 to 2 mg/L is consistent with time dependence. The PK/PD relationships of TMC207 and SQ109 are less well understood. In the present study, the activity of TMC207 continued to increase as its concentration increased up to 3 mg/L. Mean plasma concentrations of TMC207 of 1.77 mg/L have been reported after 2 weeks of 400 mg QD dosing; these decline to 0.9 mg/L during subsequent 200 mg TIW dosing [27]. Tibotec has indicated the latter dose was selected to maintain trough plasma concentrations above 0.6 mg/L. The present findings indicate higher TMC207 doses may be more efficacious, particularly when combined with PNU-100480. Additional studies are warranted to confirm this hypothesis, and to determine the safety of such higher doses. In the case of SQ109, conclusions must be considered tentative, as its killing is concentration-dependent, and its concentrations in the human lung are not yet known.

PNU-101603, the sulfoxide metabolite of PNU-100480, appeared not to contribute to its parent when the two were combined *in vitro* (figure 2); similarly, the observed cumulative WBA of PNU-100480 after oral dosing was no greater than that predicted *in vitro* based solely on concentrations of the parent. Levels of PNU-101603 reach concentrations in humans several times that of PNU-100480 [12]. Nonetheless, this finding would appear to indicate that metabolites of PNU-100480 contribute little if any to its intracellular mycobactericidal activity.

The finding that combinations of PA-824 with PNU-100480 or TMC207 were less than additive, and in some circumstances, antagonistic, was unexpected. Studies in the mouse model of other drug combinations that include PNU-100480 have not shown evidence of antagonism [28]. However, a recent study of Tasneen *et al* found antagonism when PA-824 was added to the combination of TMC207 plus pyrazinamide in the mouse model, resulting in >1 log increases in CFU counts that could not be explained by drug-drug PK interactions [29]. The basis of these observations is uncertain. PA-824 appears to act by different mechanisms depending on the microbial culture conditions [30]. When tested against aerobic, replicating cultures, it interferes with the synthesis of ketomycolates [31], which are important components of the mycobacterial cell wall. Structure-functional analyses of related nitroimidazoles indicate this activity is unrelated to that exerted against hypoxic, non-replicating cultures, which instead appears to reflect respiratory poisoning by PA-824-derived nitric oxide, with subsequent depletion of bacterial ATP [30]. Combinations of PA-824 and TMC207 may therefore be less than fully additive under hypoxic conditions, as they share a common mechanism of action. The mechanism of action of PA-824 has not been studied under the conditions present in whole blood culture, which are neither hypoxic nor fully replicating. Under non-hypoxic conditions, nitric oxide triggers a dormancy response in *M. tuberculosis* through activation of DosR [32]. Resulting alterations in replication and biosynthesis may affect the activity of other drugs such as PNU-100480 and TMC207. Further research is warranted to test this hypothesis.

These experiments were intended to provide a rapid survey of potential drug combinations rather than to serve as definitive experiments. As such, their limitations should be acknowledged. Studies of antimicrobial activity conducted by adding drugs directly to cultures eliminate the greatest source of variability, that of inter-subject differences in pharmacokinetics and metabolism. Additional population-based studies will be required to better understand how these PK differences affect the efficacy of new TB regimens. Three dimensional modeling of drug interactions [33] may provide a more accurate estimation of their combined effects than the averaging method used here. Studies of WBA in TB patients will be required to better understand the contribution of host immune mechanisms in the cultures. Lastly, since drug concentrations remain constant in each whole blood culture, post-antibiotic effects (PAE) are not expressed; these might otherwise prevent bacterial regrowth as drug levels fall below MIC *in vivo*. However, sub-MIC concentrations of PNU-100840 and TMC207 are not anticipated for the regimens of greatest interest, minimizing the significance of this shortcoming.

In summary, this study used a whole blood culture model to examine potential novel drug combinations that might ultimately be used to treat XDR-TB. The main findings were that combinations including PNU-100480, TMC207, and SQ109 were highly active and fully additive, whereas those including PA-824 were less than additive and in some instances antagonistic. Further studies of the PNU-TMC combination are warranted. The whole blood method can accelerate TB drug development.
